# Wide Awake Local Anaesthesia No Tourniquet Surgery of the Foot and Ankle: A Review of Indications, Technique, Patient Satisfaction, and Complications

**DOI:** 10.7759/cureus.74939

**Published:** 2024-12-01

**Authors:** Muhammad A Hamid, Zubair Younis, Muhammad Mannan, Zikrullah Kalim, Zafar A Khan, Rudra M Prabhu, Nayan Shrivastava, Nadia Rashid

**Affiliations:** 1 Orthopaedic Surgery, University Hospitals Birmingham, Birmingham, GBR; 2 Orthopaedic Surgery, The Royal Wolverhampton NHS Trust, Wolverhampton, GBR; 3 Anaesthesiology and Critical Care, Royal Wolverhampton NHS Trust, Wolverhampton, GBR; 4 Trauma and Orthopaedics, Worcestershire Acute Hospitals Trust, Worcestershire, GBR; 5 Trauma and Orthopaedics, University Hospitals Birmingham, Birmingham, GBR; 6 Histopathology, Government Medical College, Srinagar, Srinagar, IND

**Keywords:** ankle and foot, local anaesthetic, review article, walant, wide-awake surgery

## Abstract

Wide-awake surgery of the hand was surrounded by a lot of apprehension, mainly over concerns around using epinephrine near digits and its potential to cause digital ischemia and necrosis. With multiple reports underlining its safety and effectiveness, it is now being widely adopted in hand and wrist surgery. The British Society for Surgery of the Hand has already published guidelines on operating outside of main theatres, with an emphasis on wide awake local anaesthesia no tourniquet (WALANT). However, the same cannot be said for its use in foot and ankle procedures. There have been a handful of reports describing WALANT in bony and soft tissue procedures of the foot and ankle, with varied success. We aim to describe the scope of WALANT in these procedures and explore in detail its current role in the domain of lower extremity wide-awake surgery.

## Introduction and background

Wide awake local anaesthesia no tourniquet (WALANT), as the technique is named, implies the administration of local anaesthetic along with a suitable dilution of epinephrine in order to provide anaesthesia as well as haemostasis without the use of either conventional anaesthesia or tourniquet. With its gradual adoption in hand and wrist surgery, there have been numerous reports of its success, both in terms of patient outcomes and satisfaction, as well as its safety [1,2]. As the name suggests, the WALANT principle relies on the patient being wide awake, liberal use of local anaesthetic with adrenaline, and no tourniquet for haemostasis. The use of local anaesthetic eliminates the risk of sedation. It is cheap, time efficient and obviates the need for an anaesthetist in most cases. This serves to reduce operating theatre staffing pressures for procedures that are suitable for WALANT [3]. WALANT uses a mixture of lignocaine and adrenaline to provide anaesthesia and haemostasis in one injection. In addition to its use in reducing the operating theatre burden of elective hand/wrist surgeries, WALANT has also been described with relative success in open reduction internal fixation of distal radius fractures, metacarpal/phalangeal fractures and extensive soft tissue procedures such as spaghetti wrist reconstruction [4-6]. Lately, some authors have reported the use of WALANT in foot and ankle surgeries, especially uni-malleolar fractures, metatarsal fractures, and Achilles tendon repair [7-9]. However, the indications for WALANT in foot and ankle surgery are far less diverse and prevalent as compared to its indications in hand and wrist surgery [10].

## Review

Safety

Concerns regarding patient safety have been the biggest hindrance to the widespread adoption of the WALANT model. There has been considerable resistance to the use of WALANT in digits due to the perceived risk of digital ischemia and necrosis [11]. However, landmark reviews by Denkler and Lalonde et al. have served to dispel this notion and mark a significant step towards the adoption of epinephrine in hand surgery [12,13]. A critical analysis by Thomson and colleagues in 2007 further clarified that injection of procaine and cocaine along with epinephrine might have been the actual cause of digital necrosis [14]. This is amplified when cocaine and procaine vials are past their expiry, as the solution starts turning acidic. None of the 21 patients (between 1880 and 2000) they reported digital ischemia in had an attempt at phentolamine rescue, which has the potential to reverse iatrogenic digital ischemia [15].

In addition to providing a bloodless field during surgery, adrenaline serves to prolong the effect of lignocaine by delaying its clearance from the site of surgery by restricting blood flow. Also, by adding 1.1-1.8 ml of 8.5% sodium bicarbonate to a 10 ml mixture, the pH is brought up to between 7.38 and 7.62. This 'buffering' serves to minimize injection pain and accelerates the onset of action of local anaesthetic [16].

Concentration

The mixture of lignocaine used is usually 1% lignocaine with 1:100000 adrenaline. Some propose even more dilute solutions containing 0.25% lignocaine with 1:400000 adrenaline when a larger surface area block is required [15], while others have used concentrations of epinephrine as high as 1:40000 [8]. Care must be taken not to exceed the maximum dose of 7mg/kg of lignocaine.

Reversal

When encountered with sustained, complete blanching of the digits, which is extremely rare [17], 1 mg of phentolamine diluted in 5mL of saline may be injected into the space where epinephrine was used [15]. One can also administer phentolamine rescue to all patients with mild peripheral vascular disease following the completion of the procedure to counter the effect of epinephrine [18]. This is done by injecting 1 mg of phentolamine diluted in 5 ml of normal saline in the area where epinephrine was injected. Phentolamine has been conclusively shown to reverse the vasoconstrictive effects of locally infiltrated epinephrine [19].

Despite the overwhelming data available on the safety of WALANT in fingers and toes, manufacturer labels for adrenalized lignocaine still carry a warning that its use is contraindicated for anaesthesia of fingers, toes, tip of nose, ears, and penis [20].

Contraindications

Patients with significant peripheral vascular disease allergy to lignocaine are absolute contraindications to the use of WALANT. The current evidence suggests that significant peripheral vascular disease (PVD) is an absolute contraindication, whereas mild PVD with palpable pulse and normal capillary refill might be considered with a reduced dose of epinephrine and phentolamine rescue available. There are no guidelines for these patients, and their suitability for WALANT must be decided on a case-by-case basis. There is also evidence that a standard dose of local anaesthetic might be less effective in patients with a history of chronic opioid (mis)use [21]. Also, patients with anxiety surrounding the idea of wide-awake anaesthesia might not be suitable for the procedure [7].

Clinical benefits

WALANT provides adequate anaesthesia in surgeries of the foot and ankle; this has been demonstrated in multiple reports by various authors [7,18,22,23]. In addition to providing anaesthesia, WALANT is quite effective in providing a bloodless field during surgery [22]. Some reports have found that the total blood loss is lower following WALANT when compared to conventional anaesthesia with a tourniquet [8]. Also, it has been shown that as general anaesthesia does not block synaptic transmission of pain during surgery, it predisposes the patient to experience heightened pain in the postoperative period and an increased analgesic requirement following surgery [24]. Also, other complications of conventional anaesthesia, like postoperative nausea and vomiting, are not seen with local anaesthetic [18]. Haemostasis in WALANT surgeries relies solely on epinephrine and not on the use of a tourniquet. This avoids all the well-described local and systemic complications associated with the use of pneumatic tourniquets [25]. Wide awake surgery also allows for the assessment of adequate tension during tendon transfer procedures, as the patient can be asked to perform an active range of motion during surgery [26].

One of the benefits of WALANT is that it is administered by the surgical team. This reduces the need for anaesthetic staff and associated machinery. This affects significant cost savings. As many foot and ankle procedures are bony in nature and many require intra-operative fluoroscopy, their adoption into a more office-based approach hasn't been widely adopted as with hand and wrist surgeries, where the trend has largely moved to office-based surgery for many common conditions incorporating the concepts of field surgery and wide-awake anaesthesia [27]. Office-based needle ankle arthroscopy, however, has been performed with reasonable success [28,29].

Technique

The local anaesthetic mixture should preferably be combined as close to the time of injection as possible. Adrenalized lignocaine solutions gradually turn more acidic after reconstitution. The pain of lignocaine injections is linked to its acidic pH. Also, warmer solutions tend to cause less pain upon injections [30]. A small gauge needle (preferably 27 gauge) is used for the infiltration. A smaller needle decreases pain and limits the speed of injection, thereby reducing pain. The needle is introduced perpendicular to the skin, and a 1 cm wheal is raised [22]. After waiting for one to two minutes, the needle is advanced towards the desired areas and the whole circumference is injected. When it comes to pain during the administration of WALANT, there are studies detailing injection techniques that have been demonstrated to reduce pain during the administration of local anaesthetic. Some of the techniques employed include digital pressure around the injection site and utilizing distraction techniques to steer patients' attention away from the injection [22]. Some authors have described ways to distract patients by various means to minimize injection pain. These include listening to music or engaging in conversation [30]. Eutectic lignocaine can be applied topically to reduce the pain of needle insertion. However, they must be applied 90 to 120 minutes prior to the needle prick for the eutectic mixture to give maximum effect [31]. Using separate needles for drawing the anaesthetic mixture and for injecting avoids the use of blunted needles for the actual injection. This has been shown to significantly reduce the pain of needle insertion [32]. Tactile distraction is another tactic that can be employed to reduce the pain of needle insertion [33]. Skin stimulation by techniques such as pinching, stretching or pressing near needle insertion sites reduces the perceived painful stimulus [34]. Also, in case of lacerations or open wounds, the needle can be inserted directly into subcutaneous fat instead of penetrating the skin. This has been shown to significantly reduce the pain of injection [35]. Lastly, Strazar et al. recommend that patients be asked to score the amount of pain they felt during the process of injecting local anaesthetic. This serves to provide feedback to the surgeon and offers a great learning opportunity in order to perfect the craft of injecting local anaesthetic [30].

Indications

We have summarized the indications for WALANT in foot and ankle surgery in Table 1.

**Table 1:** Regions of the foot and ankle amenable to WALANT surgery as described by various authors WALANT - wide awake local anaesthesia no tourniquet

Location	Indication	Authors
Toes	Phalangeal fractures	Bilgetekin et al. [7]
	Phalangeal fusions	Wright et al. [18]
Foot	Metatarsal fractures	Bilgetekin et al. [7]
	Lisfranc injury	Bilgetekin et al. [7]
	Tendon repair	MacNeill et al. [23]
	Tendon transfer	MacNeill et al. [23]
	Metatarsal osteotomy	Pamuk et al. [36], Wright et al. [18], Lavigne et al. [10]
	Metatarsal fusion	Wright et al. [18]
	Tarsal fusion	MacNeill et al. [23]
	Metalwork removal	Macneill et al. [23]
Ankle	Malleolar fractures	Bilgetekin et al. [7], Li et al. [8], Borg et al. [37]
	Metalwork removal	Poggetti et al. [38], Sabaghzadeh et al. [39], Macneill et al. [23]
	Achilles repair	Wu et al. [9], Bilgetekin et al. [7]
	Needle arthroscopy	Colasanti et al. [28], Mercer et al. [29]
Miscellaneous	Distal tibia debridement	MacNeill et al. [23]

Trauma

Foot and ankle injuries form a large part of the trauma and orthopaedics emergency intake, and many injuries are amenable to management using WALANT. Bilgetekin et al. described the use of WALANT in ankle injuries [7]. They evaluated a total of 31 patients, of whom 15 had a medial malleolar fracture, five lateral malleolus, five Achilles ruptures, two proximal phalangeal fractures, one each with a Lisfranc injury, a medial malleolus fracture + syndesmotic injury, one with a deltoid ligament + syndesmotic injury, and a fifth metatarsal fracture. They prepared a 1% lignocaine mixture with 1:100000 epinephrine and 8.4% sodium bicarbonate to buffer the acidic effect of lignocaine. 

Their described mixture for making 50mL of the anaesthetic solution was 0.5mg/1mL epinephrine + 25mL of 2% lignocaine + 5mL 8.4% sodium bicarbonate + 19mL 0.9% isotonic sodium chloride. Infiltration was done into the soft tissues of the surgical area and up to the periosteum of the contralateral cortex for bony procedures. The authors gave 25-30 minutes for the effect of epinephrine to manifest before starting surgery. The Visual Analog Scale (VAS) for pain and the Visual Analog Scale for Anxiety (VAS-A) was recorded during surgery. They also recorded the incidence of postoperative complications and the duration of hospital stay. They reported a mean VAS pain score of 1 and a VAS-A score of 1 during surgery. None of their patients needed an additional rescue anaesthetic infiltration, with the mean duration of surgery being 36.6 minutes. They reported no complications in their patients either during or after surgery. They did not have a control group to compare pain scores in patients who underwent conventional anaesthesia, and their pain assessment did not extend to the postoperative period [7].

Li and colleagues enrolled a total of 18 patients whose fractures were suitable for WALANT fixation [8]. Fracture types included were isolated uni-malleolar fractures, bi-malleolar fractures, and tri-malleolar fractures not requiring fixation of the posterior malleolus. Out of the 18 patients selected, five were excluded for reasons of anxiety/nervousness or patient refusal to undergo WALANT. Thirteen patients were operated successfully under WALANT. They used 1% lignocaine but admixed this with 1:40000 epinephrine. For cases requiring syndesmotic fixation, an additional 5-10 ml of the same mixture was injected into the syndesmotic space from the anterior aspect of the fibula. Their surgeries varied from 40 minutes to 120 minutes. Two of their patients required an additional top-up during surgery due to elevating pain scores. They did not experience any local complications, nor did they notice systemic manifestations of the higher concentration of epinephrine used. No patients were given another type of anaesthesia due to the failure of WALANT. They surmised that WALANT has its own place despite the presence of various safe methods of anaesthesia for the foot and ankle, namely, spinal anaesthesia, popliteal blocks, and local anaesthesia with IV sedation. All the other methods require anaesthetists or experienced ultrasound guidance with considerable technical demands. WALANT is a cost-effective alternative that can be administered by the operating surgeons themselves.

Osseous procedures

Lavigne et al. (2024) evaluated the prospects of WALANT surgery in osteotomies of the first metatarsal in the foot [10]. They compared a total of 37 patients in the WALANT group versus 24 in the general anaesthesia group. They concluded that there was no statistically significant difference in pain levels at four hours and 24 hours between both groups. Time spent in the WALANT group was shorter, and the mean length of stay in the recovery room was also shorter, with both these differences being statistically significant.

MacNeill et al. conducted a retrospective analysis of patients undergoing various osseous and non-osseous procedures of the forefoot [23]. Patients were asked to fill out a questionnaire at an average of 6.5 months after their surgery. Out of 30 total patients, 27 responded. Eighty-seven per cent of patients indicated that they would prefer to be wide awake for another procedure, with 83% reporting that their surgery was better than their expectations. However, the authors cautioned against using this method of anaesthesia in hindfoot hardware removals as they reported greater intraoperative anxiety and pain. Due to the retrospective nature of this study and the significant potential of introducing recall bias five months after the procedure, the authors conducted another study to address those concerns. This time, the authors conducted a prospective study comparing patients undergoing forefoot surgery using wide-awake local anaesthesia with patients undergoing forefoot surgeries with general anaesthesia [18]. Two such groups comprising 20 patients each were compared against each other in terms of their reported ratings of anxiety and pain before, during, and after the procedure. Their procedures included nine first metatarsophalangeal joint fusions, four hallux valgus corrective osteotomies and three claw tow reconstructions. At the conclusion of the procedure, patients in both their treatment groups received a local infiltration of bupivacaine to help with postoperative pain relief. They concluded that WALANT patients reported lower levels of postoperative anxiety than general anaesthesia patients. They also reported lower pain levels when compared to their general anaesthesia counterparts. Both of these differences were statistically significant (p<0.001). Patients undergoing WALANT also experienced a lower incidence of postoperative nausea and vomiting. They concluded that although effective and safe, wide-awake surgery might add to the stress and difficulty experienced by the surgeon in a complex case as it requires a certain amount of interaction with the patient.

Pamuk et al., in their report on 34 patients undergoing hallux valgus correction, had 15 patients in the WALANT group and 19 patients in the traditional anaesthesia group. They reported no significant difference between the groups in terms of postoperative satisfaction, osteotomy type, and complications [36]. Visual analogue scale scoring for pain was similar between the groups during surgery and at six hours post-surgery. They, however, noted that pain during injection of anaesthetic was higher in the WALANT group compared to lumbar injection for spinal anaesthesia. They used a single 27G needle for all their infiltrations. They concluded that WALANT is a feasible alternative in hallux valgus surgery, reducing anaesthesia-related complications and cost and, at the same time, affording adequate haemostasis without the use of a tourniquet. 

Metalwork removals

Sabaghzadeh et al. conducted a randomized control trial with 30 patients in each arm for distal fibula metalwork removal. One group was administered spinal anaesthesia, and a tourniquet was used, while the other group was administered WALANT. There were no significant differences between the two groups in their VAS scores at four hours and at two weeks following surgery. However, the VAS score in the WALANT group was significantly lower the day after surgery. They also demonstrated lower blood loss in patients operated under WALANT [39].

The previously cited study by MacNeill and colleagues also reported on hardware removals of the ankle, midfoot and hindfoot. While they reported good success in ankle and midfoot, all three of their patients undergoing hindfoot hardware removals reported greater levels of anxiety and intra-operative pain. They surmised that scar tissue from previous trauma and surgery might have prevented adequate diffusion of local anaesthetic. They recommended that surgeons continue to use conventional anaesthetic techniques for hindfoot hardware removals [23].

Tendon repairs

Wu et al. explored the application of WALANT in the surgical management of Achilles tendon ruptures [9]. In their prospective randomized controlled trial, they divided 48 patients into two treatment arms. One group used the WALANT technique, while the other used epidural anaesthesia with a tourniquet for a channel-assisted minimally invasive repair (CAMIR). There was a significant difference between the study and control groups in terms of operative time and pain on day one after surgery. The WALANT group demonstrated lower operating room time and lesser hospital stay compared to the epidural anaesthetic group. Also, pain on day one was significantly less in the WALANT group. These observations are confounded, though, by the fact that a tourniquet was used in the control group, and the operative time in the control group was significantly longer than the WALANT group. There is evidence that the use of a tourniquet alone is linked to higher postoperative pain and discomfort, and this might explain the higher postoperative pain in the control group [40].

Arthroscopy

Colasanti et al. described the use of wide-awake local anaesthesia in the management of anterior ankle impingement by performing in-office needle arthroscopy (IONA). They used a combination of shavers and burrs during the procedure for performing synovial/bony resections where needed and reported minimal pain in their patients undergoing the procedure. Post-operatively, their patients reported significant symptom relief and excellent patient-reported outcomes and satisfaction. They noted three complications: late calcaneo-fibular ligament pain in one patient and nerve pain after the procedure in two patients. The exact nature of this pain was not described, but the pain was deemed 'irritating' and 'not debilitating' [28]. Another report by Mercer and colleagues studying IONA using wide-awake office-based surgery in 10 patients reported excellent patient-reported outcomes and no complications during or after surgery [29].

Barriers to adoption

The biggest hindrance to the use of WALANT has been the fear of the development of digital ischemia and necrosis. There have been many studies that serve to dispel that myth, and the safety of WALANT in digital nerve blocks has been established [11,41]. Secondly, unfamiliarity with the procedure and the fact that the patient is wide awake discourages many surgeons from trying this procedure as it can increase their own stress and anxiety related to the procedure [23]. This can be addressed by appropriate patient selection and a fully informed consent-taking process whereby the surgeon and the patient are on the same page with respect to goals, expectations, and rescue strategies available during surgery in case the effect of the anaesthetic starts wearing off [22]. Mayich and colleagues have described using a consent document for WALANT as separate from the one used for surgery, as it enabled better discussions and a better understanding of the anaesthetic process [22].

## Conclusions

The adoption of wide awake local anaesthesia no tourniquet (WALANT) represents a significant advancement in surgical practice, which is now showing promise in foot and ankle surgery. Its proven safety, cost-effectiveness, and ability to provide anaesthesia and haemostasis without the complications associated with general anaesthesia or tourniquet use are well-supported by clinical evidence. Patients benefit from reduced pain, quicker recovery times, and decreased hospital stay, while surgeons gain the ability to engage patients intraoperatively to optimize outcomes and reduce costs. However, challenges such as injection pain, patient anxiety, and surgeon unfamiliarity with the technique highlight the need for careful patient selection, a tailored consent-taking process and managing expectations. It might not be suitable in every situation, but with new literature expanding the indications for its use and establishing safety, continued research in this domain would serve to further cement its role in osseous and soft tissue procedures of the foot and ankle.
